# Effect of *n*-3 long-chain polyunsaturated fatty acid intake on the eicosanoid profile in individuals with obesity and overweight: a systematic review and meta-analysis of clinical trials

**DOI:** 10.1017/jns.2021.46

**Published:** 2021-07-21

**Authors:** Guilherme R. B. Schweitzer, Isabela N. M. S. Rios, Vivian S. S. Gonçalves, Kelly G. Magalhães, Nathalia Pizato

**Affiliations:** 1 Graduate Program in Human Nutrition, Department of Nutrition, University of Brasilia, Brasilia, Brazil; 2 Graduate Program in Public Health, University of Brasilia, Brasília, Brazil; 3 Laboratory of Immunology and Inflammation, Department of Cellular Biology, Institute of Biology, University of Brasília, Brasília, Brazil

**Keywords:** Eicosanoids, Inflammation, *n*-3 PUFA, Obesity, Systematic review

## Abstract

Dietary *n*-3 polyunsaturated fatty acids (PUFAs) present beneficial effects on counteracting inflammation status, displaying a critical anti-inflammatory role and maintaining physiological homeostasis in obesity. The primary objective of this systematic review was to evaluate the effect of *n*-3 PUFAs intake on the eicosanoid profile of people with obesity and overweight. The search strategy on Embase, Scopus, PubMed, Web of Science, Cochrane Library, Google Scholar and ProQuest was undertaken until November 2019 and updated January 2021. The effect size of *n*-3 PUFAs on prostaglandins was estimated by Glass's, type 1 in a random-effect model for the meta-analysis. Seven clinical trials met the eligible criteria and a total of 610 subjects were included in this systematic review, and four of seven studies were included in meta-analysis. The intake of *n*-3 PUFAs promoted an overall reduction in serum pro-inflammatory eicosanoids. Additionally, *n*-3 PUFAs intake significantly decreased the arachidonic acid COX-derived PG eicosanoid group levels (Glass's Δ −0⋅35; CI −0⋅62, −0⋅07, *I*^2^ 31⋅48). Subgroup analyses showed a higher effect on periods up to 8 weeks (Glass's Δ −0⋅51; CI −0⋅76, −0⋅27) and doses higher than 0⋅5 g of *n*-3 PUFAs (Glass's Δ −0⋅46; CI −0⋅72, −0⋅27). Dietary *n*-3 PUFAs intake contributes to reduce pro-inflammatory eicosanoids of people with obesity and overweight. Subgroup's analysis showed that *n*-3 PUFAs can reduce the overall arachidonic acid COX-derived PG when adequate dose and period are matched.

## Introduction

Obesity is one of the most prevalent chronic conditions presently seen in western society^(1)^ and it leads to increase activation of inflammatory signalling, characterised by a rise in eicosanoid levels^(2)^. In individuals with obesity, visceral adipose tissue presents a dysfunctional phenotype when compared with lean individuals’ tissue. Excessive adipose tissue accumulation can potentially modify phospholipase A2 (PLA2), cyclooxygenase 2 (COX-2) and 5-lipooxygenase (5-LOX) activity, thereby increasing bioactive eicosanoid mediators such as prostaglandins (PG) and leukotrienes (LT)^(3,4)^. This metabolic modification seems to determine a state of chronic low-grade inflammation, which is recognised as a critical factor for the establishment and progression of metabolic dysfunction associated with obesity^(5)^. These obesity-related comorbidities are closely linked to the presence of a persistent activation of pro-inflammatory signalling pathways in adipose tissue, which severely disrupts key metabolic checkpoints in this tissue^(5)^. The chronic pro-inflammatory eicosanoid synthesis is recognised as a key factor in the development of insulin resistance^(6)^, tissue inflammation^(7)^, renal injury^(8)^ and increased cardiovascular maladaptations^(9)^. In addition to the heightened production of inflammatory mediators, obese adipose tissue shows an intrinsic inability to resolve uncontrolled inflammation and to restore tissue homeostasis and functionality^(10)^. Titos *et al.* identified a group of genes associated with the inflammatory process that was differentially modulated in people with obesity, and COX-2 was also significantly up-regulated in their adipose tissue^(11)^. In obesity, the main problem is the imbalances in the complex network of eicosanoids synthesis, resulting from the chronicity of the inflammatory response.

Inflammation and dietary fat, especially *n*-6/*n*-3 polyunsaturated fatty acids (PUFAs) ratio, have a tight correlation;^(12)^ therefore, the composition of the cell membrane influences eicosanoid metabolism. There is enzymatic competition by the use of these PUFAs as substrates for inflammatory mediators synthesis^(2)^, and the higher *n*-6/*n*-3 PUFA ratio intake is directly associated with augmentation in COX and LOX-derived pro-inflammatory eicosanoids^(13,14)^ such as prostaglandin E2 (PGE2), leukotriene B4 (LTB4) and thromboxane A2 (TXA2), derived from *n*-6 fatty acids such as arachidonic acid (AA)^(15)^. Bowers *et al.* demonstrated in the breast tissue of women with obesity a greater macrophage COX-2 expression and more PGE2 production^(16)^. In individual with obesity and diabetes mellitus type 2, the production of pro-inflammatory AA metabolites, including PGE2, leukotrienes (LT) and HETEs, was related to insulin resistance, and PGF2α was linked to hepatic gluconeogenesis, a major driver of fasting hyperglycaemia^(17)^.

Conversely, higher plasma levels of *n*-3 PUFA are associated with reduced obesity risk^(18)^. since the intake of *n*-3 eicosapentaenoic acid (EPA) has the capability to modulate eicosanoid synthesis profile in COX and LOX-dependent pathways, generating the 3-series PG and TX, and 5-series LT and lipoxins (LX), which are less bioactive eicosanoids than the ones generated by AA^(19)^. *n*-3 docosaexaenoic acid (DHA) intake is associated with docosanoid synthesis, a similar yet different category of molecules that include resolvins, protectins, neuroprotectins and maresins, commonly referred to as specialised pro-resolvin mediators (SPMs)^(20,21)^. Because of enzymatic competition, a higher EPA and DHA consumption might shift bioactive eicosanoid synthesis from AA metabolites (pro-inflammatory activity) to EPA and DHA metabolites (less pro-inflammatory activity and even modestly anti-inflammatory activity)^(22)^. Besides COX and LOX enzyme pathway, cytochrome P450 enzyme family also synthesise eicosanoid mediators as 5-HEPE (EPA-derived) and 5-HETE (AA-derived) contributing to modulate inflammation response^(23,24)^. These findings reinforce and strengthen the importance of COX and LOX mediator's activation in the development of obesity-related complications.

Significant modifications of inflammatory signalling and serum eicosanoid profile have been seen during intervention with EPA and DHA fatty acids^(25,26)^, suggesting a protective effect of *n*-3 PUFA. A recent previous study^(27)^ showed that marine-derived *n*-3 PUFA presented a beneficial effect on reducing major pro-inflammatory eicosanoids serum values in unhealthy subjects. However, to the best of our knowledge, there has been no study conducted to summarise the available evidence of the effects of *n*-3 PUFA intake on eicosanoid, markers of inflammation, of individuals with obesity and overweight without chronic diseases. Therefore, the aim of the present study was to evaluate the effects of *n*-3 PUFA intake on a variety of eicosanoids on adults with obesity and overweight, through a systematic review and meta-analysis of controlled trials.

## Materials and methods

The current systematic review and meta-analysis was conducted as recommended by the state-of-the-art method Preferred Reporting Items for Systematic Reviews and Meta-analyses (PRISMA – Appendix 1)^(28)^, and the protocol was registered in Prospective Register of Systematic Reviews (PROSPERO – CRD42020153362).

### Information sources and search strategies

A comprehensive search was executed in the following databases: PubMed, Cochrane library, Embase, Scopus and Web of Science, and grey literature (Google Scholar and ProQuest). Publications up to 7 November 2019 were examined, and updated on 25 January 2021.

The search strategy was reviewed by an investigator with experience in systematic reviews in accordance with the Peer Review of Electronic Search Strategies (PRESS) checklist criteria^(28)^. The following strategy was adapted for databases: (‘Morbid obesity’ OR ‘Severe obesity’ OR ‘Abdominal obesity’ OR ‘Central obesity’ OR ‘Visceral obesity’ OR ‘Obese men’ OR ‘Obese women’ OR ‘Overweight’ OR ‘Overweight men’ OR ‘Overweight women’ OR ‘Excess weight’ OR ‘obese’ OR ‘obesity’ OR ‘Fat accumulation’ OR ‘fatness’ OR ‘body fatness’ for population main characteristic and combined with intervention keywords ‘*N*3 fatty acids’ OR ‘*n*-3 Fatty Acids’ OR ‘*n* 3 Fatty Acids’ OR ‘*n*3 Fatty Acids’ ‘W3 fatty acids’ OR ‘w-3 fatty acids’ OR ‘w 3 fatty acids’ OR ‘*N*3 Polyunsaturated Fatty Acid’ OR ‘*n*-3 Polyunsaturated Fatty Acid’ OR ‘*n* 3 Polyunsaturated Fatty Acid’ OR ‘*n*3 Polyunsaturated Fatty Acid’ OR ‘*n*-3 PUFA’ OR ‘*N* 3 PUFA’ OR ‘*N*3 PUFA’ OR ‘*N*-3 oils’ OR ‘*N*3 oils’ OR ‘*N* 3 oils’ OR ‘Omega 3 Fatty Acids’ OR ‘Eicosapentanoic Acid’ OR ‘omega 3 Eicosapentaenoic Acid’ OR ‘omega-3-Eicosapentaenoic Acid’ OR ‘Timnodonic Acid’ OR ‘Docosahexenoic Acid’ OR ‘omega 3 Docosahexenoic Acid’ OR ‘Docosahexaenoate’ OR ‘alpha Linolenic Acid’ OR ‘Linolenate’ OR ‘Linolenic Acid’ OR ‘EPA and DHA supplementation’ OR EPA OR DHA OR ‘omega 3’ OR ‘omega-3’ OR ‘fish oil’ OR ‘arachidonic acid’ OR ‘arachidonate’) AND (‘eicosatetraenoic acid’ OR eicosanoid OR Icosanoid OR Prostanoid OR Lipoxin OR Prostaglandin OR Thromboxane OR Leukotriene OR ‘hydroxyeicosatetraenoic acid’ OR ‘Isoprostane’ OR ‘dinoprostone’). The Google search was limited to the first 200 most relevant articles. No filters on language, publication date or status were applied to the results found in each database. More information about search strategies is provided in Appendix 2.

A consultation was carried out on the ClinicalTrials.gov Database portal (U.S. National Library of Medicine) in order to verify if there was any on-going or non-published article that could be included in our systematic review. Reference lists of included records were manually reviewed to identify potential studies not retrieved from databases.

Study selection was undertaken independently by two reviewers (G. S. and I. R.). The duplicates were removed, and the screening procedure was applied using Rayyan software^(29)^. Both authors (G. S. and I. R.) independently assessed the full text of preselected studies for eligibility. Disagreements were resolved by consensus and another author (V. S. S. G.) if required.

### Eligibility criteria

Clinical trials studies that were conducted on adults with obesity and overweight (more than 18 years old and less than 65 years old) were eligible. The intervention criteria were *n*-3 PUFA intake either through oral supplements or foods.

Exclusion criteria were as follows: (1) studies with subjects that underwent bariatric/metabolic surgery; (2) consensus, management, reviews, letters, conference abstracts, editorials; (3) studies evaluating subjects with inflammatory and chronic diseases; (4) use of non-steroidal anti-inflammatory drugs; and (5) absent eicosanoid measurement or measurements other than serum or plasma eicosanoids.

### Data extraction

Data were extracted undertaken independently by two investigators (G. S. and I. R.), with discrepancies resolved through group discussion. The extracted information was categorised as follows: (1) Author, Year of publication, and Country; (2) Study design; (3) Study Period; (4) *n*-3 PUFA source; (5) Study Protocol; (6) Food or supplement adherence protocol; (7) Age; (8) Baseline BMI; (9) Baseline and Post-Intervention serum eicosanoids markers; and (10) Main results.

### Risk of bias in individual studies

The risk of bias of included articles was performed according to the Joanna Briggs Institute Critical Appraisal Tools. A 9-question checklist (‘Checklist of quasi-experimental studies’) was used to assess risk of bias in non-randomised clinical trials and a 13-question checklist (‘Checklist of randomised controlled trials’) in order to evaluate randomised clinical trials. The tool consisted of 9 or 13 questions, always answered as ‘yes’, ‘no’, ‘unclear’ or ‘not applicable’. In the present study, the risk of bias was considered low when all items were answered as ‘yes’. If any item was classified as ‘no’, a high risk of bias was expected. The risk of bias of each included study was independently certified by two reviewers (G. S. and I. R.).

### Summary measures and synthesis of results

The primary outcomes were the identified measures of association between the *n*-3 PUFA intake and serum eicosanoids concentration for qualitative analysis.

Due to available data collected regarding the primary outcomes, we were able to conduct a meta-analysis to investigate the effect size of *n*-3 PUFA intake on PG concentrations. For this, we built a random-effect model using the restricted maximum-likelihood (REML) method^(30)^. The random-effect meta-analysis approach incorporates an assumption that the different studies are estimating different, yet related, intervention effects^(31,32)^.

The difference between the parameters investigated from baseline to end point was estimated by Glass's Δ type 1 with its respective 95 % CI^(33)^. The Glass's Δ is suitable for studies whose comparison with a control group is not possible^(34)^. In studies with more than one intervention group, the highest *n*-3 PUFA dose group was considered for analysis. In the study that evaluated salmon and cod intake, only the salmon group was included for analysis since salmon provided seven times more *n*-3 PUFA when compared to cod. For the study with a crossover design, mean changes between the levels of markers at the end of two intervention periods were used according to Cochrane Handbook^(35)^.

Heterogeneity of treatment effects between studies was tested using the *χ*^2^ method (*P* < 0⋅10) and its magnitude using *I*^2^. When *I*^2^ was less than 40 %, it was not considered important, according to Cochrane's collaboration recommendation^(36)^. In order to investigate parameters influencing heterogeneity, we performed subgroup analyses, considering the following strata: doses of *n*-3 (higher or lower than 0⋅5 g EPA + DHA *n*-3 PUFA as recommended by ISSFAL for the general adult population^(37)^); source of administration of *n*-3 (food or oral supplement) and intervention time (more than 8 weeks or up to 8 weeks). Due to the small number of studies included in the meta-analysis, it was not possible to perform meta-regression and analysis of publication bias^(32)^.

## Results

### Selection of relevant studies

**Fig. 1.**
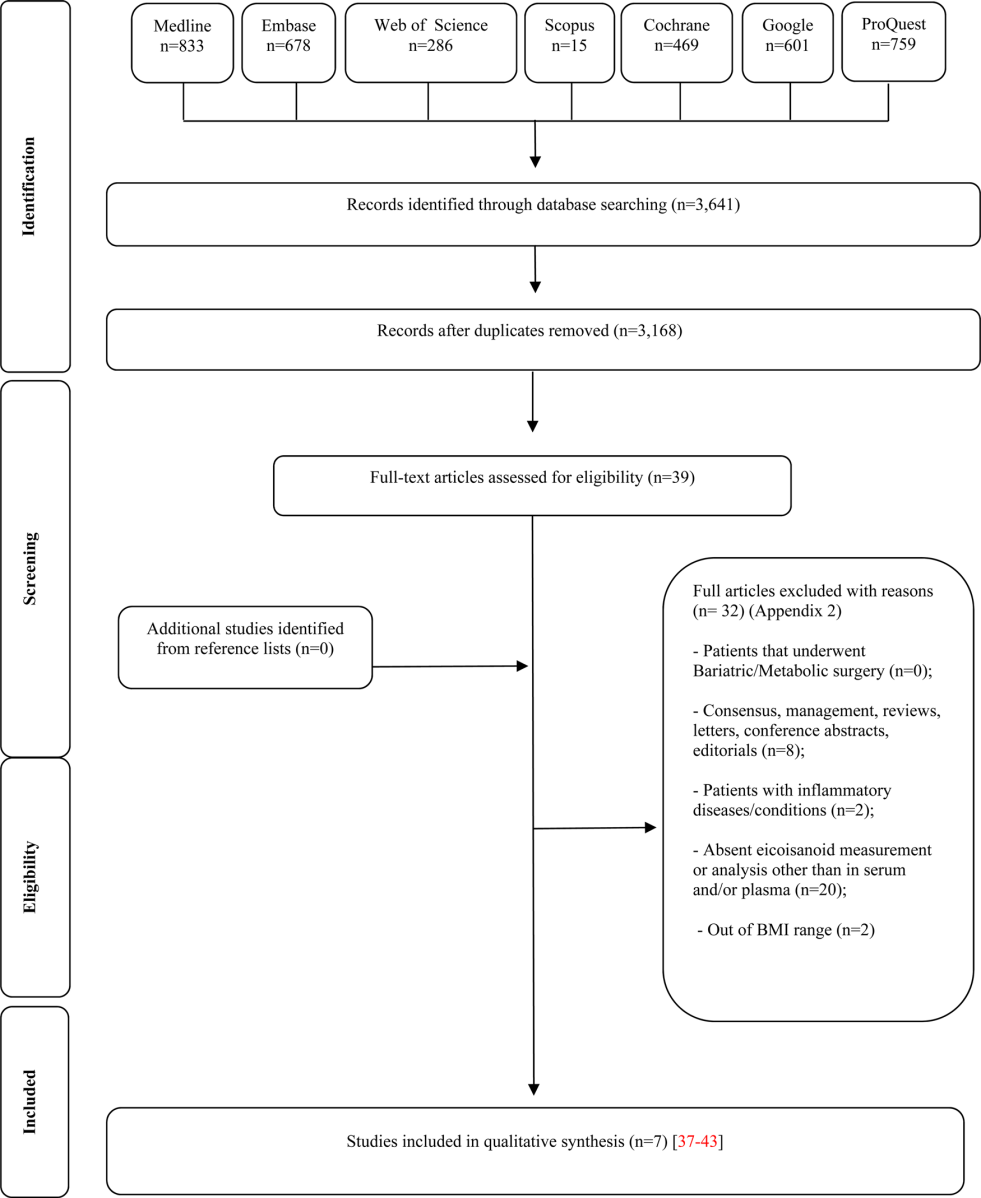
Flow diagram of studies evaluated in the review (adapted from PRISMA).

The initial search identified a total of 3641 articles from 7 databases, and after removing duplicates, 39 potential studies met the eligibility assessment and complete full-text reading. Thirty-two articles were excluded and the reasons are presented in Appendix 3. At the end, seven articles^(38–44)^ were selected for this systematic review. After selection, the full reference list of each article was checked in order to identify possible additions and no article was potentially eligible. In addition, the *Clinicaltrials.gov* registers were consulted and no protocol associated with eligible articles has been identified. A flow diagram of the screening process is shown in Fig. 1.

Seven studies were selected for qualitative analysis. Of these, three studies were excluded from meta-analysis because the required data were not available. One study did not analyse PG, one was excluded because only provided figures without quantitative information, and one present insufficient information (no data at the baseline in control groups). We contacted the authors for data but received no replies.

### Characteristics of included studies

The characteristics of the studies included are summarised in Table 1. Seven clinical trials included 610 individuals with obesity and/or overweight published between 2010^(44)^ and 2019^(38)^ (Table 1). Among them, six studies^(39–44)^ are randomised trials and only one study^(38)^ is a non-randomised trial. The studies were conducted in different countries, including Spain^(38,40)^, Germany^(39)^, Denmark^(41)^ and Poland^(43)^. Ramel *et al.* was a multicenter study that included Spain, Iceland and Ireland^(44)^.

**Table 1.** Summary of descriptive characteristics and outcomes of interest of the included studies (*n* 7)

Author, Year and Country	Study Design	Study Period	*n*-3 PUFA Source	Study Protocol	Food or supplement adherence protocol	Age (mean ± sd)	Baseline BMI (kg/m^2^)	Marker	*n*-3 or *n*-6 fatty acid family	*n*-3 Intervention group (mean ± sd)		Significance*
										Baseline values (pg/ml)	Post-intervention values (pg/ml)	
Celada, 2019 – Spain^(38)^	Non-randomised crossover clinical trial	4 weeks	Enriched frankfurters and pâtés	***n*-3 Intervention group (*n* 18)**: 15**⋅**5 % total fat; 2 g of ALA plus EPA plus DHA/d **Control group (*n* 18):** 18 % total fat for frankfurters and 30 % total fat for patés)	Seventy-two-hour dietary registers	Both groups: 44**⋅**9 ± 10**⋅**3	Both groups: 28**⋅**6 ± 2**⋅**5	6-keto-PGF1α TXB2	*n*-6 *n*-6	747 ± 452 309 ± 158	563 ± 336 254 ± 75**⋅**7	***P* < 0⋅001** ***P* < 0⋅05**
Dawczynski, 2013 – Germany^(39)^	Placebo-controlled, randomised double-blind parallel clinical trial	10 weeks	Enriched yoghurt	***n*-3 Intervention group (*n* 16):** 5**⋅**5 % total fat; 1**⋅**59 g EPA/d, 1**⋅**12 g DHA/d, 0**⋅**07 g ALA/d and 0**⋅**23 g DPA/d **Control group** **(*n* 14):** 3**⋅**5 % total fat	Food Frequency Protocol	**Intervention group:** 61**⋅**82 ± 7**⋅**13 **Control group:** 58**⋅**23 ± 7**⋅**38	**Intervention group:** 25**⋅**91 ± 3**⋅**67 **Control group:** 26**⋅**14 ± 3**⋅**87	5-HEPE 8-HEPE 9-HEPE 11-HEPE 12-HEPE 15-HEPE 18-HEPE 5-HETE 8-HETE 9-HETE 11-HETE 12-HETE 15-HETE 15-HETrE PGE3 PGE2 PGD2 PGE1 TXB2 LTB4	*n*-3 *n*-3 *n*-3 *n*-3 *n*-3 *n*-3 *n*-3 *n*-6 *n*-6 *n*-6 *n*-6 *n*-6 *n*-6 *n*-6 *n*-3 *n*-6 *n*-6 *n*-3 *n*-6 *n*-6	6**⋅**16 ± 6**⋅**80 0**⋅**20 ± 0**⋅**21 0**⋅**44 ± 0**⋅**38 0**⋅**22 ± 0**⋅**19 0**⋅**76 ± 0**⋅**82 0**⋅**29 ± 0**⋅**26 0**⋅**58 ± 0**⋅**41 22**⋅**68 ± 19**⋅**96 0**⋅**97 ± 0**⋅**77 0**⋅**82 ± 0**⋅**90 0**⋅**89 ± 0**⋅**78 2**⋅**92 ± 2**⋅**70 1**⋅**26 ± 0**⋅**97 0**⋅**32 ± 0**⋅**28 0**⋅**01 ± 0**⋅**00 0**⋅**07 ± 0**⋅**05 0**⋅**52 ± 0**⋅**52 0**⋅**06 ± 0**⋅**07 0**⋅**06 ± 0**⋅**08 0**⋅**18 ± 0**⋅**39	3**⋅**19 ± 4**⋅**02 0**⋅**18 ± 0**⋅**24 0**⋅**31 ± 0**⋅**30 0**⋅**21 ± 0**⋅**23 1**⋅**01 ± 0**⋅**83 0**⋅**37 ± 0**⋅**38 0**⋅**70 ± 0**⋅**73 11**⋅**60 ± 27**⋅**82 0**⋅**57 ± 1**⋅**16 0**⋅**53 ± 1**⋅**18 0**⋅**52 ± 1**⋅**29 2**⋅**24 ± 1**⋅**62 0**⋅**87 ± 1**⋅**68 0**⋅**21 ± 0**⋅**47 0**⋅**08 ± 0**⋅**19 0**⋅**06 ± 0**⋅**07 0**⋅**29 ± 0**⋅**79 0**⋅**03 ± 0**⋅**06 0**⋅**09 ± 0**⋅**11 0**⋅**07 ± 0**⋅**05	*P* = 0**⋅**063 *P* = 0**⋅**646 *P* = 0**⋅**284 *P* = 0**⋅**570 *P* = 0**⋅**312 *P* = 0**⋅**507 *P* = 0**⋅**406 ***P* = 0⋅030** ***P* = 0⋅030** ***P* = 0⋅041** ***P* = 0⋅030** *P* = 0**⋅**305 *P* = 0**⋅**422 *P* = 0**⋅**053 ***P* = 0⋅008** *P* = 0**⋅**148 ***P* = 0⋅041** *P* = 0**⋅**213 *P* = 0**⋅**397 *P* = 0**⋅**176
DeLuis, 2016 – Spain^(40)^	Single-blinded, randomised, controlled, prospective clinical trial	24 weeks	Oral oil supplement	***n*-3 Intervention group (*n* 14):** DHA 0**⋅**5 g/d during the first 60 d and 0**⋅**25 g/d till 180 d **Control group (*n* 15):** placebo capsules with the same scheme (composition not mentioned)	Not informed	**Intervention group:** 47**⋅**4 ± 9**⋅**1 **Control group:** 44**⋅**3 ± 11**⋅**7	**Intervention group:** 33**⋅**4 ± 1**⋅**4 **Control group:** 32**⋅**95 ± 1**⋅**9	15-HETE 12-HETE 8-HETE 5-HETE TXB2 PGE2 LTB4 PD1	*n*-6 *n*-6 *n*-6 *n*-6 *n*-6 *n*-6 *n*-6 *n*-3	23**⋅**76 ± 38**⋅**35 5359**⋅**26 ± 3431**⋅**47 57**⋅**85 ± 51**⋅**38 369**⋅**63 ± 106**⋅**82 131**⋅**26 ± 95**⋅**62 0**⋅**14 ± 0**⋅**34 Not detectable 3**⋅**80 ± 5**⋅**27	72**⋅**95 ± 51**⋅**50 3226**⋅**87 ± 1431**⋅**22 82**⋅**88 ± 36**⋅**02 369**⋅**10 ± 149**⋅**68 148**⋅**01 ± 71**⋅**79 13**⋅**31 ± 15**⋅**46 16**⋅**31 ± 18**⋅**99 6**⋅**31 ± 3**⋅**03	***P* < 0⋅05** ***P* < 0⋅05** ***P* < 0⋅05** *P* > 0**⋅**05 *P* > 0**⋅**05 ***P* < 0⋅05** ***P* < 0⋅05**^b^ *P* > 0**⋅**05
Nielsen, 2012 – Denmark^(41)^	Parallel double-blinded randomised controlled clinical trial	6 weeks	Oral oil supplement	***n*-3 Intervention group (*n* 25):** 0**⋅**64 g EPA/d and 0**⋅**48 g DHA/d **Control group (*n* 25):** 2 g of olive oil/d	Food Frequency Questionnaire	**Intervention group:** 58**⋅**0 ± 7**⋅**4 **Control group:** 55**⋅**4 ± 9**⋅**5	**Intervention group:** 30**⋅**8 ± 4**⋅**2 **Control group:** 29**⋅**5 ± 3**⋅**3	5-HETE^a^ 5-HEPE LTB4 LTB5	*n*-6 *n*-3 *n*-6 *n*-3	350 ± 18 58 ± 6 240 ± 12 9 ± 1	328 ± 18 117 ± 6 205 ± 12 14 ± 1	***P* = 0⋅46**^b^ ***P* < 0⋅001**^b^ ***P* = 0⋅26**^b^ ***P* = 0⋅005**^c^
O'Sullivan, 2014 – United States^(42)^	Double-blind, placebo controlled randomised clinical trial	6 weeks	Oral oil supplement	***n*-3 Intervention group (*n* 28):** 5 g fish oil with 2 g EPA/d and 1 g DHA/d **Control group (*n* 42):** 5 g soyabean oil/d	Food Frequency Questionnaires	**Intervention group:** 37**⋅**2 ± 12 **Control group:** 34**⋅**1 ± 12	**Intervention group:** 27**⋅**0 ± 4**⋅**3 **Control group:** 27**⋅**7 ± 4**⋅**6	5-HEPE^d^ LTB4^e^	*n*-3 *n*-6	Slope = 11, *r*^2^ 0**⋅**55 (*n* 28) Slope = −2**⋅**0, *r*^2^ 0**⋅**25 (*n* 29)		***P* < 0⋅0001** ***P* = 0⋅005**
Polus, 2016 – Poland^(43)^	Randomised placebo-controlled double-blind clinical trial	12 weeks	Oral oil supplement	***n*-3 Intervention group (*n* 24):** 1**⋅**29 g DHA and 0**⋅**27–0**⋅**45 g EPA/d **Control group (*n* 35):** Not informed	No adherence protocol was informed	**Intervention group:** 45**⋅**9 ± 9**⋅**3 **Control group:** 47**⋅**3 ± 12	**Intervention group:** 34**⋅**4 ± 2**⋅**69 **Control group:** 34**⋅**7 ± 3	LXA4 LXA5	*n*-6 *n*-3	50**⋅**29 ± 19**⋅**10 62**⋅**3 ± 24**⋅**38	57**⋅**63 ± 17**⋅**83 79**⋅**8 ± 31**⋅**16	*P* = 0**⋅**069 *P* = 0**⋅**058
Ramel, 2010 – Iceland, Spain and Ireland^(44)^	Randomised, controlled dietary intervention trial	8 weeks	Salmon and oral oil supplement	**Food intervention group (*n* 84):** 150 g salmon, 3 times per week. 2**⋅**1 g LC *n*-3 PUFA **Fish oil intervention group (*n* 80):** 1**⋅**3 g EPA plus DHA (6 cápsules per day) **Control group (*n* 80):** no seafood (6 sunflower oil capsules per day)	Food Frequency Questionnaire	**Food intervention group** Male: 31**⋅**6 ± 5**⋅**6 Female: 30**⋅**9 ± 5**⋅**0 **Fish oil intervention group** Male: 31**⋅**0 ± 5**⋅**9 Female: 30**⋅**9 ± 5**⋅**0 **Control group** Male: 32**⋅**6 ± 4**⋅**9 Female: 31**⋅**7 ± 5**⋅**6	**Food intervention group** Male: 30**⋅**5 ± 1**⋅**3 Female: 30**⋅**3 ± 1**⋅**5 **Fish oil intervention group** Male: 29**⋅**5 ± 1**⋅**2 Female: 30**⋅**1 ± 1**⋅**7 **Control group** Male: 30**⋅**1 ± 1**⋅**5 Female: 29**⋅**9 ± 1**⋅**5	PGF2	*n*-6	Salmon Male: 188 ± 182 Female: 202 ± 199 Fish oil Male: 270 ± 451 Female: 228 ± 321	Salmon Male: 170 ± 197 Female: 148 ± 184 Fish oil Male: 189 ± 141 Female: 139 ± 199	***P* < 0⋅05**^f^ ***P* < 0⋅05**^f^

RF, reduced fat; EPA, eicosapentaenoic acid; DHA, docosahexaenoic acid; PG, prostaglandin; LT, leukotriene; *n*-3 PUFAs, omega-3 polyunsaturated fatty acids; 6-keto-PGF2α, 6-keto-prostaglandin F2 alpha; PGI2, prostacyclin *I*^2^; HODE, hydroxyoctadecaenoic; HEPE, hydroxy eicosapentaenoic acid; HETE, hydroxyeicosatetraenoic acid; HETrE, 15-hydroxyeicosatrienoic acid; LXA, lipoxin A; TXB2, thromboxane B2; LtB4, leukotriene B4; LA, linoleic acid; ALA, α-linolenic acid; LC-PUFA, long-chain polyunsaturated fatty acid; LtB5, leukotriene B5; LOX, lipooxygenase enzymes; COX, cyclooxygenase enzymes; CYP450, cytochrome P450.

a All results are presented in pg/ml, except for Nielson *et al.* (ng/10^7^ cells) and O'Sullivan *et al*. with association analysis.

b Compared with the control group.

c Compared within *n*-3 intervention groups.

d 5-HEPE (nM) *v.* erythrocytes EPA (mol%) Linear regression analyses.

e LTB4 (nM) *v.* erythrocytes EPA (mol%) Linear regression analyses.

f Significant before–after differences when the data were viewed for all subjects together.

* *P*-values without letters represent significant difference or not from within groups. The *P*-value was extracted from the data of articles included in this review.

The bold values in (Significance column) represent the *p* values that presented a significant result (*p* < 0.05).

Generally, participants were middle-aged and overweight. The lower baseline BMI was 25⋅91 ± 3⋅67^(39)^ and the higher BMI was 34⋅4 ± 2⋅69^(43)^. The mean age ranged from 31 ± 5⋅9 years old^(44)^ to 61⋅82 ± 7⋅13^(39)^. The majority of studies analysed both men and women^(39–42,44)^, except by Celada *et al.* and Polus *et al.*, which included only men and only women, respectively. The intervention period ranged from 4^(38)^ to 24 weeks^(40)^.

The *n*-3 PUFA content of the protocol interventions was provided by oral oil supplements^(40–44)^ or by food such as *n*-3 enriched frankfurters and patés^(38)^, *n*-3 enriched yoghurt^(39)^ and salmon^(44)^. *n*-3 enriched food was provided as a mixture of different *n*-3 long-chain PUFA family, including EPA, DHA, DPA (docosapentaenoic acid)^(38)^ and ALA (alpha-linolenic acid)^(39)^. One study supplemented individuals using exclusively DHA fatty acid capsules^(40)^ and three other studies supplemented with EPA plus DHA capsules^(41–43)^. Only one study presented the total fat amount and did not mention which specific fatty acids were included in the intervention protocol^(44)^. The control group were supplemented with olive oil^(41)^, maize/soyabean oil^(42)^ or sunflower oil capsules^(44)^ and one study did not specify which oil was used^(43)^. Studies that utilised food as PUFAs source presented conventional fruit yoghurt^(39)^ and normal-fat frankfurters and pates as control groups^(38)^.

The lower *n*-3 PUFA dose observed was 0⋅25 g for DHA^(40)^ and the higher dose was 3 g for a combination of ALA, EPA, DPA and DHA^(39,42)^.

Each study measured a particular subset of eicosanoids, varying from leukotrienes, PG, thromboxanes, lipoxins to PUFA metabolites such as HEPEs and HETEs. Five of seven studies measured both pro- and anti-inflammatory eicosanoids^(39–43)^, and two studies measured only the pro-inflammatory ones^(38,44)^.

### Adherence protocols and analysis of cellular fatty acid content

Different protocols were used in each of the studies in order to verify adherence to the intervention. Food frequency questionnaires^(39,41,42,44)^ and Dietary Register^(38)^ were used. Two studies did not mention the specific adherence protocol used^(40,43)^.

Five of seven studies analysed the fatty acid content after the intervention period, four of them used erythrocytes membrane^(39,40,42,43)^, one used neutrophils membrane^(41)^, and all of them used gas chromatography (GC) as the standard methodology to identify the fatty acid profile. All studies showed that *n*-3 PUFA intake led to a significant membrane fatty acid incorporation, suggesting compliance with the *n*-3 PUFA intervention protocol.

### Risk of bias within individual studies

The Joanna Briggs Institute appraisal tools were used to assess risk of bias for all studies included. Of the six RCTs, two were judged to be at ‘low risk of bias’^(39,41)^. The non-randomised clinical trial^(38)^ and four RCTs^(40,42–44)^ were classified as being at ‘high risk of bias’.

**Fig. 2.**
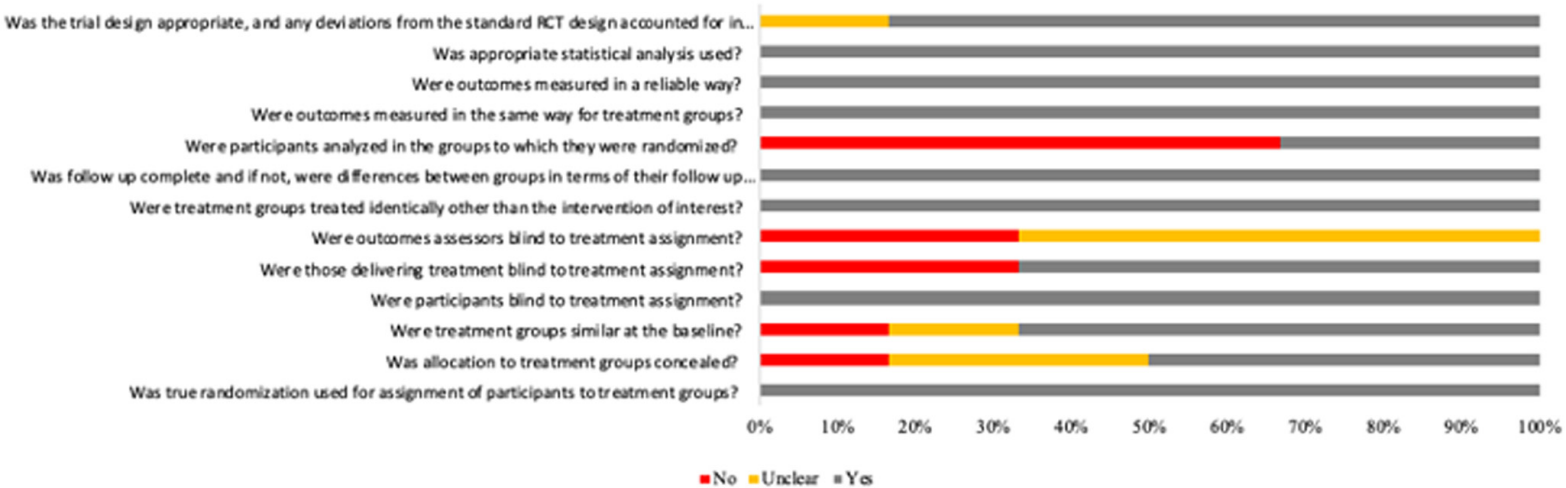
Review of judgements of authors about each risk of bias item according to The Joanna Briggs Institute Critical Appraisal Checklist for Randomised Controlled Trials is presented as percentages across all included studies.

The included studies reported low risks of bias regarding randomisation, blindness, outcomes measured. Considering the randomised trials, 100 % indicated a low risk of bias for randomisation of participants to the treatment group, blinding of participants to treatment assignment, identical groups other than the intervention of interest, complete follow-up, outcome measured in an equal and reliable way for treatment groups and appropriate statistical analysis. In all RCTs assessed, the one domain judged with a total high risk for bias was regarding intention-to-treat analysis, resulting in one ‘unclear’^(41)^ and six ‘no’ answers^(39,40,42–44)^. In two RCTs, individuals delivering the treatment and outcome assessors were not blind to treatment assignment^(40,44)^. In only one RCT, the allocation of treatment groups was not concealed^(44)^. In all trials assessed, there was at least one domain judged with unclear risk for bias. Fig. 2 shows the individual risk of bias assessments for all studies included in this work.

### Results of individual studies

Five of seven studies presented an overall reduction in pro-inflammatory eicosanoids after *n*-3 PUFA intervention, and a less pronounced effect in anti-inflammatory eicosanoids (Table 1). Only DeLuis *et al.*^(34)^ showed enhanced serum levels in AA-derived eicosanoids levels after *n*-3 PUFA intake.

COX-derived eicosanoids such as 6-keto-prostaglandin F1α (6-keto-PGF1α)^(38)^, PGE2^(39,40)^, prostaglandin D2 (PGD2)^(39)^, prostaglandin F2 (PGF2)^(44)^ and TXB2^(38)^ presented lower serum levels after *n*-3 PUFA intervention. DeLuis *et al.*^(40)^ was the only study that presented opposite effects with an increase in PGE2 and TXB2 levels after EPA plus DHA supplementation. The eicosanoid prostaglandin E3 (PGE3) presented reduced serum levels after *n*-3 enriched yoghurt^(39)^.

Concerning the LOX-5-derived eicosanoids, lower serum levels of LTB4 were observed after *n*-3 PUFA supplementation in one study^(41)^, but interestingly DeLuis *et al.*^(40)^ observed higher levels after the intervention period. The HETE family such as 5-HETE, 8-HETE, 9-HETE, 11-HETE^(39)^ and 12-HETE^(40)^ showed reduced serum levels after *n*-3 PUFA intake. However, 15-HETE and 8-HETE presented increased serum levels after *n*-3 PUFA intervention in the study conducted by DeLuis *et al.*^(40)^. In only one study, 5-HEPE EPA-derived eicosanoid presented higher serum levels after intervention^(41)^.

### Synthesis of results

**Fig. 3.**
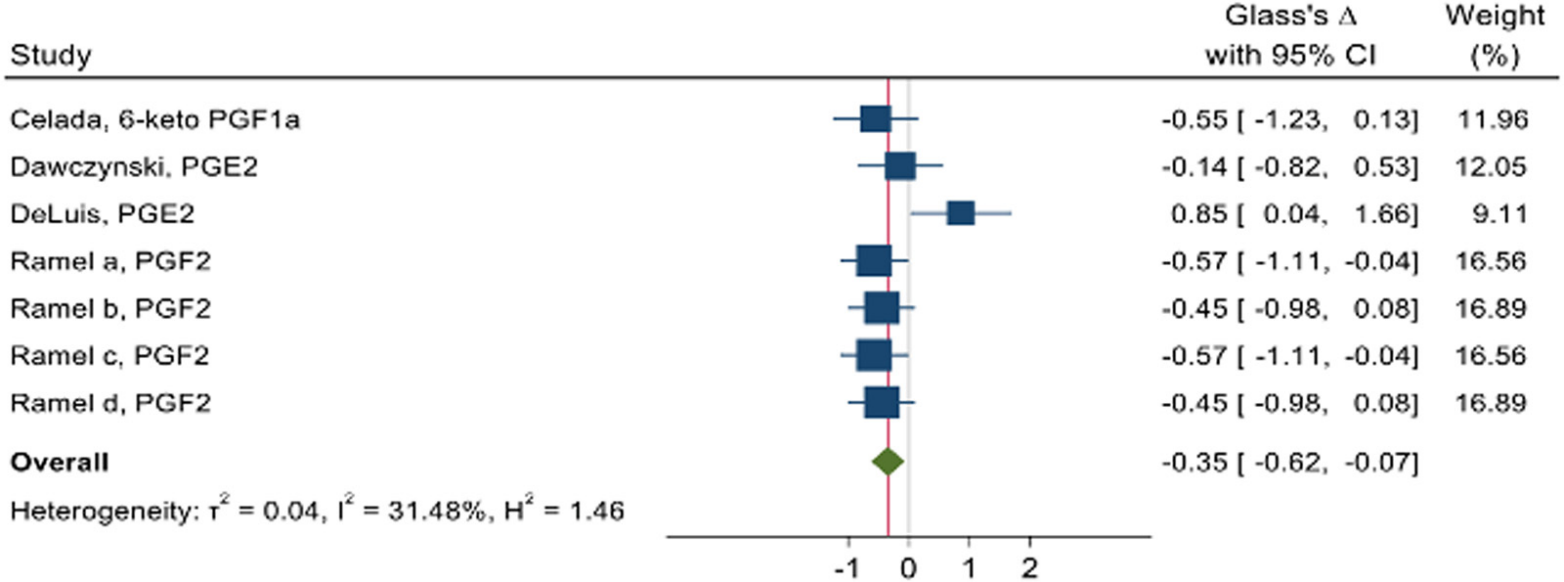
Pooled effect size of *n*-3 PUFA intake on COX-derived prostaglandins markers in individuals with obesity and overweight. Pooled effect estimates from meta-analysis are expressed as standardised mean differences (SMD), represented by diamonds. The 95 % CIs is the line through the diamond and were estimated with the use of a generic inverse variance random-effect model. Interstudy heterogeneity was detected with the use of Cochran's *Q* statistic and quantified with the use of the *I*^2^ statistic. 6-keto-PGF1α, 6-keto-prostaglandin F1alpha; PGE2, prostaglandin E2; PGF2, prostaglandin F2. *n*-3 sources: Celada: *n*-3 enriched paté group; Dawczynski: *n*-3 enriched yoghurt group; DeLuis: DHA supplementation group; Ramel a: fish oil male group; Ramel b: fish oil female group; Ramel c: salmon male group; Ramel d: salmon female group.

Due to the available data, we were able to conduct a subgroup analysis with the arachidonic acid COX-derived PG eicosanoid group. Meta-analysis presented an overall reduction in PG series (Glass's Δ −0⋅35; 95 % CI −0⋅62, −0⋅07) after *n*-3 PUFA intake (Fig. 3).

### Subgroup analysis

Subgroup analysis showed significant effects by reducing arachidonic acid COX-derived PG levels when *n*-3 PUFA was consumed in higher doses (Glass's Δ −0⋅46; 95 % CI −0⋅71, −0⋅21) and with the period of intervention up to 8 weeks (Glass's Δ −0⋅35; 95 % CI −0⋅62, −0⋅07). There was no difference in arachidonic acid COX-derived PG levels when food (Glass's Δ −0⋅34; 95 % CI −0⋅82, 0⋅13) or oil supplement (Glass's Δ −0⋅31; 95 % CI −0⋅73, 0⋅12) was taken into consideration. More details are shown in Table 2.

**Table 2.** Subgroup analysis for the effect of and *n*-3 PUFA intake on COX-derived prostaglandins profile on subjects with obesity and overweight

Subgroups	*N*	Glass's Δ	95 % CI	*I*^2^ (%)	*P* (*χ*^2^)
Overall	7	–0⋅35	–0⋅62, −0⋅07	31⋅48	–
*n*-3 PUFA dose					
High	5	–**0**⋅**46**	–**0⋅72,** −**0⋅27**	0⋅00	0⋅88
Low	2	0⋅13	–1⋅24, 1⋅50	85⋅16	0⋅01
Time of intervention					
More than 8 weeks	2	0⋅33	–0⋅65, 1⋅30	70⋅74	0⋅06
Up to 8 weeks	5	–**0**⋅**51**	–**0⋅76,** –**0⋅27**	0⋅00	0⋅99
*n*-3 PUFA source					
Food	2	–0⋅34	–0⋅83, 0⋅13	0⋅00	0⋅41
Oil Supplement	5	–0⋅31	–0⋅73, 0⋅12	63⋅17	0⋅04

PUFA, polyunsaturated fatty acid; CI, confidence interval.

The bold values in (Significance column) represent the *p* values that presented a significant result (*p* < 0.05).

## Discussion and conclusions

The studies presented in this systematic review suggest the anti-inflammatory effects of *n*-3 PUFA intake on serum eicosanoid profile and, for the first time, in individuals with obesity and overweight. The meta-analysis result showed an overall reduction on arachidonic acid COX-derived PG serum levels. Also, a reduction was seen in doses higher than 0⋅5 g/d of EPA + DHA and with the period of intervention less than 8 weeks. This is an important finding considering that *n*-3 PUFA could improve metabolic alterations by mediating chronic low-grade inflammation related to obesity.

The daily supply of *n*-3 PUFAs either by supplementation or enriched food by diet are recommended by several agencies and organisations since previous studies suggest their protective effect on cardiovascular diseases^(45,46)^ and cancer^(47,48)^. The World Health Organization (WHO, 2000) recommends 1–2 % of daily energy consumption from ômega-3 PUFA for the general population and the International Society for the Studies of Fatty Acids and Lipids^(37)^ recommend at least 500 mg/d of EPA + DHA for the general adult population aiming for cardiovascular health. However, there is no current daily recommendation for adults with obesity and overweight, but it is noticed that the excess of fat, especially the visceral fat, increases risk for cardiovascular diseases and other comorbidities^(49,50)^. Actually, the ratio of dietary *n*-6 to *n*-3 PUFA, rather than the absolute amount of *n*-3 PUFA, is important in determining the development of inflammatory eicosanoid response. Based on the understanding that *n*-6 PUFA induces a more potent inflammatory response, whereas *n*-3 PUFA are thought to have a less potent inflammatory effect, the fluctuation in *n*-6/*n*-3 ratio intake contributes to the eicosanoid profile release from adipose tissues. When pro-inflammatory eicosanoids are enhanced, they can unbalance the inflammatory signal and promote the recruitment of M1-polarized macrophages, increasing pro-inflammatory adipokines and cytokines secretion by adipose tissue^(46,47)^. Given the inefficiency of transformation from dietary ALA to *n*-3 long-chain PUFA^(19)^, increasing dietary consumption of EPA and DHA helps to reach the maximum beneficial effects, reducing the inflammatory response in individuals with obesity^(51,52)^.

Investigation about mechanisms underlying the attenuation of inflammatory response and metabolic dysfunction in individuals with obesity by *n*-3 PUFA intake are progressing over the last decades. Anti-inflammatory effects of *n*-3 PUFA are primarily demonstrated by two main mechanisms: inhibitory secretion of pro-inflammatory mediators and greatly reduced macrophage migration into adipose tissue. The effect in obese animal models (high-fat diet-induced obesity and genetic obesity) is promising, but the proposed mechanisms still require further confirmation in humans. Evidence from *in vitro* and *in vivo* studies suggests that EPA and DHA can reduce pro-inflammatory adipokine synthesis, decreasing the inflammatory crosstalk between adipocytes and infiltrative immune cells^(26)^, murine macrophages^(53)^ and mice CD8 lymphocytes^(54)^. In adipocytes, the peroxisome proliferator-activated receptor gamma (PPARγ) functions as an effector of T helper 2 cytokines (Th2), whose activation is necessary for the differentiation of circulating monocytes into M2 macrophages and transcription of anti-inflammatory genes. PUFA *n*-3 substitution of AA in adipocyte membrane phospholipids results in a decrease in the level of PGE2 and a subsequent reduction in the enzymatic activity of fatty acid synthesis^(55)^, which restricts adipocyte hypertrophy. On the other hand, treatment with *n*-3 PUFA results in a significant decrease in the size of mature adipocytes and accumulation of smaller adipocytes in obese diabetic mice^(56)^, and the decrease in the formation of PGD2 and its derivatives, known as PPARγ ligands, explains the effects of *n*-3 PUFAs on the proliferation and maturation of adipocytes^(57)^. However, the exact mechanisms of the interaction of *n*-3 PUFA effects between immune cells and adipocytes need to be explored.

White adipocytes have an important role in the orchestrating inflammatory response in white adipose tissue, by releasing several pro-inflammatory molecules and activating and recruiting immune cells^(58)^. The COX-1 and COX-2 enzymes are involved in the inflammation regulatory process^(53,54)^. Thus, a pro-inflammatory microenvironment leads to a higher release of pro-inflammatory eicosanoids from AA, especially from the prostaglandin family. The breast tissue of women with obesity was demonstrated that a higher concentration of pro-inflammatory cytokines promotes greater macrophage COX-2 expression and produces more PGE2^(59)^. The present study clearly links obesity and low-grade chronic inflammation, processes mediated by COX-2 and aromatase expression in human breast tissue.

Studies have demonstrated that *n*-3 PUFA may replace AA and shift the eicosanoid profile, but distinct results have been found depending on which type of *n*-3 fatty acid is being offered to the studied population. *In vitro* study showed that EPA and DHA replaced 25–50 % of AA in phospholipids on macrophage cellular membrane, and the AA fatty acids replace significantly reduced 50–65 % the PGE2, TXB2 and 6-keto-PGF1α synthesis^(55)^. The study conducted by Nielsen and colleagues with a duration of 6 weeks offered an oral supplementation of both EPA and DHA and found significant reductions in LTB4 serum levels when subjects in the intervention group was compared with their respective controls. These results were also seen in individuals with obesity with EPA 0⋅64 g/d and DHA 0⋅48 g/d supplementation^(35)^ and with EPA 2 g/d and DHA 1 g/d in overweight subjects^(36)^, which are considered low and high doses, respectively. Interestingly, the study conducted by De Luis *et al.*^(34)^ presented contrasting results regarding eicosanoids synthesis. It revealed an increase in LTB4, 8-HETE and 15-HETE after 0⋅5 g/d DHA supplementation for 6 months concomitantly with a very low-calorie ketogenic diet. According to the authors, adipose tissue is considered as a source of NEFA and fatty acid-derived bioactive inflammatory lipid mediators and during the intervention period of significant weight loss, these may be released from adipose tissue to systemic circulation. Also, it needs to be considered that the protocol supplementation was composed solely by DHA (did not include EPA), which may influence the eicosanoid profile synthesis, since DHA is a major precursor of docosanoids, a similar but distinct subset of biomolecules involving in the inflammatory response.

Evaluating the PG group, meta-analysis presented a significant reduction in arachidonic acid COX-derived PG when intake was higher than 0⋅5 g/d EPA + DHA, regardless. This is in accordance with previous studies that stated a dose-dependent immunological response between *n*-3 intake and PGE2 and PGE3 synthesis^(21,60)^. There is also evidence in humans, suggesting that an intake between 1⋅35 and 2⋅7 g *n*-3 PUFA would be required to affect PGE2 production by mononuclear cells in healthy younger and older men^(60)^. Despite the non-significant effect from the source of *n*-3 PUFA, provided by a food source or oil supplement, it is known the better bio-utilisation of lipids from foods can be attributed to the larger amount of fat as part of the natural composition of food, favouring lipid absorption and conferring a higher bioavailability^(61)^.

Time is a critical factor when it comes to *n*-3 PUFA effectiveness, since the benefits over inflammation appear to be dependent on the incorporation of fatty acids in the cell membrane^(62)^ and that process might take about 3–4 weeks to reach its peak^(63)^. It is important to mention that the studies included in meta-analysis started from a 4-week period intervention, which comprehends sufficient time to incorporate *n*-3 fatty acids on the cell membrane. Unexpectedly, a significant reduction in arachidonic acid COX-derived PG was showed when the time of intervention was less than 8 weeks, a pattern that could not be seen for more prolonged periods (more than 8 weeks). That outcome can be associated with the different doses used in each protocol. It means that higher doses for shorter periods could elicit a significant effect on the eicosanoid profile, whereas lower doses for more prolonged periods could not. Studies measuring PG after *n*-3 PUFA supplementation in periods shorter than 8 weeks are scarce. Tecklenburg-Lund *et al.*^(64)^ showed that 3⋅2 g EPA plus 2⋅0 g DHA oral supplementation taken daily for 3 weeks was effective in reducing 11β-prostaglandin F2 in asthmatic individuals. However, a reasonable amount of evidence shows beneficial effects of *n*-3 PUFAs for more prolonged periods, with reductions in PG being observed even at the third^(65,66)^ and sixth month^(67)^ of intervention.

One of the challenges to understand the effects of *n*-3 PUFA in the inflammatory response in obesity is related to the wide variety of *n*-3 PUFA intervention protocols used in human trials. Those include dose, source of *n*-3 PUFA, distribution and amounts of total and subtypes fatty acids in the *n*-3 PUFA source, population evaluated, genetic backgrounds, environmental conditions, quality and quantity of the diet, etc^(68)^. Besides that, the baseline values of anti-inflammatory eicosanoids may mask the real effect of PUFA supplementation. Also, individual responsiveness to *n*-3 fatty acids should be taken into account as another major limitation to assess the effect on the inflammatory process and to suggest an optimal dose for *n*-3 PUFA.

The present study has strengths, including (i) an effort was made to search for data in seven different databases and rigorously following PRISMA directions in order to minimise publication bias; (2) utilisation of validated tools to characterise included studies in terms of methodological quality; and (3) the summarised pool analysis focused on studies measuring comparable outcomes with similar protocols, reducing methodological heterogeneity. Additionally, there are limitations in the present study. Firstly, our meta-analysis results used the delta values within the same group, and not between control and intervention groups. Secondly, the different types of *n*-3 fatty acids could be observed in intervention groups, and that may influence the final results, since there is a discrepant rate of interconversion between ALA, EPA, DPA and DHA fatty acids^(69)^. Lastly, due to the limited trials, the present study included a small number of clinical trials (*n* 7), and therefore, more well-conducted controlled trials are required to strengthen our findings and explore optimal doses and period intake in individuals with overweight and obesity.

In conclusion, this systematic review and meta-analysis evidence the effect of *n*-3 PUFA intake through diet or supplementation on eicosanoid synthesis, suggesting a benefit reduction on arachidonic acid COX-derived PG in individuals with obesity and overweight. Finally, *n*-3 PUFA intake may be an interesting strategy to be considered by healthcare professionals in association with a multifaceted approach to manage obesity such as a healthy eating pattern, achieving and maintaining a healthy weight and getting regular physical activity, in order to soften low-grade chronic inflammatory response in obesity treatment.

## Appendix 1. PRISMA Checklist

**Table:**

Section/topic	#	Checklist item	Reported on page #
TITLE			
Title	1	Identify the report as a systematic review, meta-analysis, or both.	1
ABSTRACT			
Structured summary	2	Provide a structured summary including, as applicable: background; objectives; data sources; study eligibility criteria, participants, and interventions; study appraisal and synthesis methods; results; limitations; conclusions and implications of key findings; systematic review registration number.	1–2
INTRODUCTION			
Rationale	3	Describe the rationale for the review in the context of what is already known.	2–3
Objectives	4	Provide an explicit statement of questions being addressed with reference to participants, interventions, comparisons, outcomes, and study design (PICOS).	3
METHODS			
Protocol and registration	5	Indicate if a review protocol exists, if and where it can be accessed (e.g. Web address), and, if available, provide registration information including registration number.	4
Eligibility criteria	6	Specify study characteristics (e.g. PICOS, length of follow-up) and report characteristics (e.g. years considered, language, publication status) used as criteria for eligibility, giving rationale.	4–5
Information sources	7	Describe all information sources (e.g. databases with dates of coverage, contact with study authors to identify additional studies) in the search and date last searched.	4–6
Search	8	Present full electronic search strategy for at least one database, including any limits used, such that it could be repeated.	4, Figure 1 and Supplementary Appendix S1
Study selection	9	State the process for selecting studies (i.e. screening, eligibility, included in systematic review, and, if applicable, included in the meta-analysis).	4–6, Supplementary Appendix S1
Data collection process	10	Describe method of data extraction from reports (e.g. piloted forms, independently, in duplicate) and any processes for obtaining and confirming data from investigators.	5
Data items	11	List and define all variables for which data were sought (e.g. PICOS, funding sources) and any assumptions and simplifications made.	5–6
Risk of bias in individual studies	12	Describe methods used for assessing risk of bias of individual studies (including specification of whether this was done at the study or outcome level), and how this information is to be used in any data synthesis.	6
Summary measures	13	State the principal summary measures (e.g. risk ratio, difference in means).	6–7
Synthesis of results	14	Describe the methods of handling data and combining results of studies, if done, including measures of consistency (e.g. *I*^2^) for each meta-analysis.	6–7
Risk of bias across studies	15	Specify any assessment of risk of bias that may affect the cumulative evidence (e.g. publication bias, selective reporting within studies).	7
Additional analyses	16	Describe methods of additional analyses (e.g. sensitivity or subgroup analyses, meta-regression), if done, indicating which were pre-specified.	6–7, Table 2 and Figure 3
RESULTS			
Study selection	17	Give numbers of studies screened, assessed for eligibility, and included in the review, with reasons for exclusions at each stage, ideally with a flow diagram.	7
Study characteristics	18	For each study, present characteristics for which data were extracted (e.g. study size, PICOS, follow-up period) and provide the citations.	7–8, Table 1
Risk of bias within studies	19	Present data on risk of bias of each study and, if available, any outcome level assessment (see Item 12).	9, Figure 2
Results of individual studies	20	For all outcomes considered (benefits or harms), present, for each study: (i) simple summary data for each intervention group, (ii) effect estimates and confidence intervals, ideally with a forest plot.	9–10
Synthesis of results	21	Present results of each meta-analysis done, including confidence intervals and measures of consistency.	10, Figure 3
Risk of bias across studies	22	Present results of any assessment of risk of bias across studies (see Item 15).	–
Additional analysis	23	Give results of additional analyses, if done (e.g. sensitivity or subgroup analyses, meta-regression [see Item 16]).	10, Table 2
DISCUSSION			
Summary of evidence	24	Summarize the main findings including the strength of evidence for each main outcome; consider their relevance to key groups (e.g. healthcare providers, users and policy makers).	9
Limitations	25	Discuss limitations at the study and outcome level (e.g. risk of bias), and at the review level (e.g. incomplete retrieval of identified research, reporting bias).	10–14
Conclusions	26	Provide a general interpretation of the results in the context of other evidence, and implications for future research.	14
FUNDING			
Funding	27	Describe sources of funding for the systematic review and other support (e.g. supply of data); role of funders for the systematic review.	15

From Moher D, Liberati A, Tetzlaff J, Altman DG, The PRISMA Group (2009) Preferred Reporting Items for Systematic Reviews and Meta-Analyses: The PRISMA Statement. *PLoS Med* 6(7), e1000097. doi:10.1371/journal.pmed1000097.

## Appendix 2. Search strategy databases

**Table:**

Pubmed	((‘Morbid obesity’[All Fields] OR ‘Morbid obesities’[All Fields] OR ‘Severe obesity’[All Fields] OR (‘obesity, morbid’[MeSH Terms] OR (‘obesity’[All Fields] AND ‘morbid’[All Fields]) OR ‘morbid obesity’[All Fields] OR (‘severe’[All Fields] AND ‘obesities’[All Fields])) OR ‘Abdominal obesities’[All Fields] OR ‘Abdominal obesity’[All Fields] OR ‘Central obesities’[All Fields] OR ‘Central obesity’[All Fields] OR ‘Visceral obesity’[All Fields] OR (‘obesity, abdominal’[MeSH Terms] OR (‘obesity’[All Fields] AND ‘abdominal’[All Fields]) OR ‘abdominal obesity’[All Fields] OR (‘visceral’[All Fields] AND ‘obesities’[All Fields])) OR ‘Obese men’[All Fields] OR ‘Obese women’[All Fields] OR ‘Overweight’[All Fields] OR ‘Overweight men’[All Fields] OR ‘Overweight women’[All Fields] OR ‘Excess weight’[All Fields] OR ‘obese’[All Fields] OR ‘obesity’[All Fields] OR ‘Fat accumulation’[All Fields] OR ‘fatness’[All Fields] OR ‘body fatness’[All Fields]) AND (‘*N*3 fatty acids’[All Fields] OR ‘*n*-3 Fatty Acids’[All Fields] OR ‘*n* 3 Fatty Acids’[All Fields] OR ‘*n*3 Fatty Acids’[All Fields] AND ‘W3 fatty acids’[All Fields] OR ‘w-3 fatty acids’[All Fields] OR ‘w 3 fatty acids’[All Fields] OR ‘*N*3 Polyunsaturated Fatty Acid’[All Fields] OR ‘*n*-3 Polyunsaturated Fatty Acid’[All Fields] OR ‘*n* 3 Polyunsaturated Fatty Acid’[All Fields] OR ‘*n*3 Polyunsaturated Fatty Acid’[All Fields] OR ‘*n*-3 PUFA’[All Fields] OR ‘*N* 3 PUFA’[All Fields] OR ‘*N*3 PUFA’[All Fields] OR ‘*N*-3 oils’[All Fields] OR (‘fatty acids, omega-3’[MeSH Terms] OR (‘fatty’[All Fields] AND ‘acids’[All Fields] AND ‘omega-3’[All Fields]) OR ‘omega-3 fatty acids’[All Fields] OR (‘*n*3’[All Fields] AND ‘oils’[All Fields])) OR ‘*N* 3 oils’[All Fields] OR ‘Omega 3 Fatty Acids’[All Fields] OR ‘Eicosapentanoic Acid’[All Fields] OR ‘omega 3 Eicosapentaenoic Acid’[All Fields] OR ‘omega-3-Eicosapentaenoic Acid’[All Fields] OR ‘Timnodonic Acid’[All Fields] OR ‘Docosahexenoic Acid’[All Fields] OR ((‘fatty acids, omega-3’[MeSH Terms] OR (‘fatty’[All Fields] AND ‘acids’[All Fields] AND ‘omega-3’[All Fields]) OR ‘omega-3 fatty acids’[All Fields] OR ‘omega 3’[All Fields]) AND Docosahexenoic[All Fields] AND (‘acids’[MeSH Terms] OR ‘acids’[All Fields] OR ‘acid’[All Fields])) OR ‘Docosahexaenoate’[All Fields] OR ‘alpha Linolenic Acid’[All Fields] OR ‘Linolenate’[All Fields] OR ‘Linolenic Acid’[All Fields] OR ‘EPA and DHA supplementation’[All Fields] OR EPA[All Fields] OR DHA[All Fields] OR ‘omega 3’[All Fields] OR ‘omega-3’[All Fields] OR ‘fish oil’[All Fields] OR ‘arachidonic acid’[All Fields] OR ‘arachidonate’[All Fields] OR ‘eicosatetraenoic acid’[All Fields])) AND ((‘eicosanoids’[MeSH Terms] OR ‘eicosanoids’[All Fields] OR ‘eicosanoid’[All Fields]) OR (‘eicosanoids’[MeSH Terms] OR ‘eicosanoids’[All Fields] OR ‘icosanoid’[All Fields]) OR (‘prostaglandins’[MeSH Terms] OR ‘prostaglandins’[All Fields] OR ‘prostanoid’[All Fields]) OR (‘lipoxins’[MeSH Terms] OR ‘lipoxins’[All Fields] OR ‘lipoxin’[All Fields]) OR (‘prostaglandins’[MeSH Terms] OR ‘prostaglandins’[All Fields] OR ‘prostaglandin’[All Fields]) OR (‘thromboxanes’[MeSH Terms] OR ‘thromboxanes’[All Fields] OR ‘thromboxane’[All Fields]) OR (‘leukotrienes’[MeSH Terms] OR ‘leukotrienes’[All Fields] OR ‘leukotriene’[All Fields]) OR ‘hydroxyeicosatetraenoic acid’[All Fields] OR ‘Isoprostane’[All Fields] OR ‘dinoprostone’[All Fields])
Web of science	(‘Morbid obesity’ OR ‘Severe obesity’ OR ‘Abdominal obesity’ OR ‘Central obesity’ OR ‘Visceral obesity’ OR ‘Obese men’ OR ‘Obese women’ OR ‘Overweight’ OR ‘Overweight men’ OR ‘Overweight women’ OR ‘Excess weight’ OR ‘obese’ OR ‘obesity’ OR ‘Fat accumulation’ OR ‘fatness’ OR ‘body fatness’) AND TÓPICO: (‘*N*3 fatty acids’ OR ‘*n*-3 Fatty Acids’ OR ‘*n* 3 Fatty Acids’ OR ‘*n*3 Fatty Acids’ ‘W3 fatty acids’ OR ‘w-3 fatty acids’ OR ‘w 3 fatty acids’ OR ‘*N*3 Polyunsaturated Fatty Acid’ OR ‘*n*-3 Polyunsaturated Fatty Acid’ OR ‘*n* 3 Polyunsaturated Fatty Acid’ OR ‘*n*3 Polyunsaturated Fatty Acid’ OR ‘*n*-3 PUFA’ OR ‘*N* 3 PUFA’ OR ‘*N*3 PUFA’ OR ‘*N*-3 oils’ OR ‘*N*3 oils’ OR ‘*N* 3 oils’ OR ‘Omega 3 Fatty Acids’ OR ‘Eicosapentanoic Acid’ OR ‘omega 3 Eicosapentaenoic Acid’ OR ‘omega-3-Eicosapentaenoic Acid’ OR ‘Timnodonic Acid’ OR ‘Docosahexenoic Acid’ OR ‘omega 3 Docosahexenoic Acid’ OR ‘Docosahexaenoate’ OR ‘alpha Linolenic Acid’ OR ‘Linolenate’ OR ‘Linolenic Acid’ OR ‘EPA and DHA supplementation’ OR EPA OR DHA OR ‘omega 3’ OR ‘omega-3’ OR ‘fish oil’ OR ‘arachidonic acid’ OR ‘arachidonate’ OR ‘eicosatetraenoic acid’) AND TÓPICO: (eicosanoid OR Icosanoid OR Prostanoid OR Lipoxin OR Prostaglandin OR Thromboxane OR Leukotriene OR ‘hydroxyeicosatetraenoic acid’ OR ‘Isoprostane’ OR ‘dinoprostone’)
Scopus	TITLE-ABS-KEY ((‘Morbid obesity’ OR ‘Morbid obesities’ OR ‘Severe obesity’ OR ‘Severe obesities’ OR ‘Abdominal obesities’ OR ‘Abdominal obesity’ OR ‘Central obesities’ OR ‘Central obesity’ OR ‘Visceral obesity’ OR ‘Visceral obesities’ OR ‘Obese men’ OR ‘Obese women’ OR ‘Overweight’ OR ‘Overweight men’ OR ‘Overweight women’ OR ‘Excess weight’ OR ‘obese’ OR ‘obesity’ OR ‘Fat accumulation’ OR ‘fatness’ OR ‘body fatness’ AND ‘*N*3 fatty acids’ OR ‘*n*-3 Fatty Acids’ OR ‘*n* 3 Fatty Acids’ OR ‘*n*3 Fatty Acids’ ‘W3 fatty acids’ OR ‘w-3 fatty acids’ OR ‘w 3 fatty acids’ OR ‘*N*3 Polyunsaturated Fatty Acid’ OR ‘*n*-3 Polyunsaturated Fatty Acid’ OR ‘*n* 3 Polyunsaturated Fatty Acid’ OR ‘*n*3 Polyunsaturated Fatty Acid’ OR ‘*n*-3 PUFA’ OR ‘*N* 3 PUFA’ OR ‘*N*3 PUFA’ OR ‘*N*-3 oils’ OR ‘*N*3 oils’ OR ‘*N* 3 oils’ OR ‘Omega 3 Fatty Acids’ OR ‘Eicosapentanoic Acid’ OR ‘omega 3 Eicosapentaenoic Acid’ OR ‘omega-3-Eicosapentaenoic Acid’ OR ‘Timnodonic Acid’ OR ‘Docosahexenoic Acid’ OR ‘omega 3 Docosahexenoic Acid’ OR ‘Docosahexaenoate’ OR ‘alpha Linolenic Acid’ OR ‘Linolenate’ OR ‘Linolenic Acid’ OR ‘EPA and DHA supplementation’ OR epa OR dha OR ‘omega 3’ OR ‘omega-3’ OR ‘fish oil’ OR ‘arachidonic acid’ OR ‘arachidonate’ OR ‘eicosatetraenoic acid’ AND eicosanoid OR eicosanoid OR prostanoid OR lipoxin OR prostaglandin OR thromboxane OR leukotriene OR ‘hydroxyeicosatetraenoic acid’ OR ‘Isoprostane’ OR ‘dinoprostone’))
Embase	(‘morbid obesity’ OR ‘severe obesity’ OR ‘abdominal obesity’ OR ‘central obesity’ OR ‘visceral obesity’ OR ‘obese men’ OR ‘obese women’ OR ‘overweight’ OR ‘overweight men’ OR ‘overweight women’ OR ‘excess weight’ OR ‘obese’ OR ‘obesity’ OR ‘fat accumulation’ OR ‘fatness’ OR ‘body fatness’) AND ((‘*n*-3 fatty acids’ OR ‘*n* 3 fatty acids’ OR ‘*n*3 fatty acids’) AND ‘w3 fatty acids’ OR ‘w-3 fatty acids’ OR ‘w 3 fatty acids’ OR ‘*n*-3 polyunsaturated fatty acid’ OR ‘*n* 3 polyunsaturated fatty acid’ OR ‘*n*3 polyunsaturated fatty acid’ OR ‘*n*-3 pufa’ OR ‘*n* 3 pufa’ OR ‘*n*3 pufa’ OR ‘*n*-3 oils’ OR ‘*n*3 oils’ OR ‘*n* 3 oils’ OR ‘omega 3 fatty acids’ OR ‘eicosapentanoic acid’ OR ‘omega 3 eicosapentaenoic acid’ OR ‘omega-3-eicosapentaenoic acid’ OR ‘timnodonic acid’ OR ‘docosahexenoic acid’ OR ‘omega 3 docosahexenoic acid’ OR ‘docosahexaenoate’ OR ‘alpha linolenic acid’ OR ‘linolenate’ OR ‘linolenic acid’ OR ‘epa and dha supplementation’ OR epa OR dha OR ‘omega 3’ OR ‘omega-3’ OR ‘fish oil’ OR ‘arachidonic acid’ OR ‘arachidonate’ OR ‘eicosatetraenoic acid’) AND (eicosanoid OR icosanoid OR prostanoid OR lipoxin OR prostaglandin OR thromboxane OR leukotriene OR ‘hydroxyeicosatetraenoic acid’ OR ‘isoprostane’ OR ‘dinoprostone’)
Cochrane	Title Abstract Keyword ‘Morbid obesity’ OR ‘Severe obesity’ OR ‘Abdominal obesity’ OR ‘Central obesity’ OR ‘Visceral obesity’ OR ‘Obese men’ OR ‘Obese women’ OR ‘Overweight’ OR ‘Overweight men’ OR ‘Overweight women’ OR ‘Excess weight’ OR ‘obese’ OR ‘obesity’ OR ‘Fat accumulation’ OR ‘fatness’ OR ‘body fatness’ in Title Abstract Keyword AND ‘*N*3 fatty acids’ OR ‘*n*-3 Fatty Acids’ OR ‘*n* 3 Fatty Acids’ OR ‘*n*3 Fatty Acids’ ‘W3 fatty acids’ OR ‘w-3 fatty acids’ OR ‘w 3 fatty acids’ OR ‘*N*3 Polyunsaturated Fatty Acid’ OR ‘*n*-3 Polyunsaturated Fatty Acid’ OR ‘*n* 3 Polyunsaturated Fatty Acid’ OR ‘*n*3 Polyunsaturated Fatty Acid’ OR ‘*n*-3 PUFA’ OR ‘*N* 3 PUFA’ OR ‘*N*3 PUFA’ OR ‘*N*-3 oils’ OR ‘*N*3 oils’ OR ‘*N* 3 oils’ OR ‘Omega 3 Fatty Acids’ OR ‘Eicosapentanoic Acid’ OR ‘omega 3 Eicosapentaenoic Acid’ OR ‘omega-3-Eicosapentaenoic Acid’ OR ‘Timnodonic Acid’ OR ‘Docosahexenoic Acid’ OR ‘omega 3 Docosahexenoic Acid’ OR ‘Docosahexaenoate’ OR ‘alpha Linolenic Acid’ OR ‘Linolenate’ OR ‘Linolenic Acid’ OR ‘EPA and DHA supplementation’ OR EPA OR DHA OR ‘omega 3’ OR ‘omega-3’ OR ‘fish oil’ OR ‘arachidonic acid’ OR ‘arachidonate’ OR ‘eicosatetraenoic acid’ in Title Abstract Keyword AND eicosanoid OR Icosanoid OR Prostanoid OR Lipoxin OR Prostaglandin OR Thromboxane OR Leukotriene OR ‘hydroxyeicosatetraenoic acid’ OR ‘Isoprostane’ OR ‘dinoprostone’ in Title Abstract Keyword – (Word variations have been searched)

## Appendix 2. Excluded articles and reasons for exclusion (*n* 32)

**Table:**

Author, Year	Reason for exclusion
Aronson *et al.* (2011)	3
Allaire *et al.* (2016)	5
Denzlinger *et al.* (1995)	5
Djuric *et al.* (2017)^(4)^	5
Gammelmark *et al.* (2012)	5
Huerta *et al.* (2014)	5
Holt *et al.* (2017)	5
Lang *et al.* (2019)^(3)^	5
Murphy *et al.* (2007)	5
Newman *et al.* (2014)	5
Peres *et al.* (2018)	5
Petersson *et al.* (2010)	5
Pickens *et al.* (2015)	5
Shearer *et al.* (2018)	5
Trebble *et al.* (2004)	3
Young *et al.* (2011)	5
Celada *et al.* (2014)	2
Bohm *et al.* (2013)	5
Itariu *et al.* (2012)	2
Fisk *et al.* (2018)	2
Lengfelder *et al.* (2016)	2
Quach *et al.* (2017)	2
Uach *et al.* (2017)	2
Hill *et al.* (2007)	5
Gruslova *et al.* (2017)	2
Pickens *et al.* (2017)^(2)^	5
Qin *et al.* (2015)	6
Nieman *et al.* (2012)	5
Kaatz *et al.* (2004)	6
Garcia *et al.* (2016)	5
Garcia-Ravelo *et al.* (2018)	5
Brenner *et al.* (2017)	2
Stephensen *et al.* (2011)	5

Exclusion criteria were as follows: (1) absence of outcomes about nutritional status/growth/quality of life; (2) qualitative study and (3) consensus/management/reviews/letters/conference abstracts/editorials.

(1) Patients that underwent bariatric/metabolic surgery;

(2) Consensus, management, reviews, letters, conference abstracts, editorials;

(3) Patients with inflammatory diseases;

(4) Anti-inflammatory drugs or supplements; and

(5) Measurements of eicosanoids other than serum and plasma out of BMI range.
